# Implications for the design of a Diagnostic Decision Support System (DDSS) to reduce time and cost to diagnosis in paediatric shoulder instability

**DOI:** 10.1186/s12911-021-01446-5

**Published:** 2021-02-27

**Authors:** Fraser Philp, Alice Faux-Nightingale, Sandra Woolley, Ed de Quincey, Anand Pandyan

**Affiliations:** 1 School of Allied Health Professions, Keele University, Keele, UK; 2 School of Pharmacy and Bioengineering, Keele University, Keele, UK; 3 School of Computing and Mathematics, Keele University, Keele, UK

**Keywords:** Physiotherapy, Shoulder instability, User centred design, Clinical decision support system, Paediatrics

## Abstract

**Background:**

Currently the diagnosis of shoulder instability, particularly in children, is difficult and can take time. These diagnostic delays can lead to poorer outcome and long-term complications. A Diagnostic Decision Support System (DDSS) has the potential to reduce time to diagnosis and improve outcomes for patients. The aim of this study was to develop a concept map for a future DDSS in shoulder instability.

**Methods:**

A modified nominal focus group technique, involving three clinical vignettes, was used to elicit physiotherapists decision-making processes.

**Results:**

Twenty-five physiotherapists, (18F:7 M) from four separate clinical sites participated. The themes identified related to ‘Variability in diagnostic processes and lack of standardised practice’ and ‘Knowledge and attitudes towards novel technologies for facilitating assessment and clinical decision making’.

**Conclusion:**

No common structured approach towards assessment and diagnosis was identified. Lack of knowledge, perceived usefulness, access and cost were identified as barriers to adoption of new technology. Based on the information elicited a conceptual design of a future DDSS has been proposed. Work to develop a systematic approach to assessment, classification and diagnosis is now proposed.

*Trial Registraty* This was not a clinical trial and so no clinical trial registry is needed.

## Background

Shoulder instability is an umbrella term used to describe complete or partial dislocation of the shoulder joint [1]. Shoulder instability can affect children between eight and 18 years but this occurs most frequently in children aged between 14 to 16 years (incidence of 164.4 /100,000 person years), [2]. Time to a confirmed diagnosis is normally two years and children can have up to 11 episodes of instability before formal diagnosis [3]. Between 70 to 90% of children have repeated dislocations and have an increased risk of early onset of shoulder arthritis [1, 3]. Shoulder instability is associated with pain, decreased movement and limited function. A plausible reason for the poor prognosis is inaccurate diagnosis which may result in inappropriate treatment selection which occurs despite the availability of multiple classification systems [4–10], diagnostic/assessment guidelines [11, 12] and management/treatment pathways [10, 13]

There is a need to improve diagnostic accuracy and prevent the development of long-term complications for this patient group [1, 2]. Healthcare services are increasingly drawing upon technological solutions to improve diagnostic accuracy and efficiency, particularly within the context of the COVID-19 pandemic and subsequent ‘Rebuilding of the NHS’ strategy [14]. One method of achieving improved diagnostic accuracy with technology is through using clinical or diagnostic decision support systems (CDSS/DDSS). The purpose of a DDSS is to provide clinicians with intelligently filtered information, specific to the patient, which may facilitate decision making, such as clinical guidelines, alerts, or diagnostic support through suggestions of differential diagnosis or narrowing of etiologic causes [15, 16]. Although, DDSSs are becoming increasingly common in the management of musculoskeletal conditions [17], existing systems are unable to support the diagnosis of shoulder instability [18].

A useful and robust DDSS should draw upon existing state of the art clinical decision making processes which subsequently inform treatment allocation. Successful implementation and adoption into clinical practice requires that the DDSS is developed in partnership with the end user group through early stakeholder involvement [19]. The aim of this study was to elicit the types of information used to make clinical decisions, with the long-term goal of designing and developing appropriate decision support technologies for the assessment and management of children with shoulder instability.

## Methods

Ethical approval was gained from University Research Ethics Committee Review (NS-190032). Participants from across four separate clinical sites were recruited within their capacity as health care professionals (physiotherapists), who have specialist interest in paediatric shoulder instability. A modified nominal focus group technique [20] comprised of the following stages was used:

- The methodology was explained to participants who were then introduced to a series of three clinical vignettes^1^ (Table 1) (original layout presented in Additional file 1).

**Table 1:** Clinical vignettes and seed questions used to elicit clinical decision-making processes

Vignettes	Seed questions
**Vignette 1** *Subjective assessment* Patient is a 16-year-old female presenting with worsening right shoulder pain. Recurrent episodes of instability/ partial shoulder displacement for the last 6 years. Not sure about the direction of instability. Competitive netball and swimming since age 12 with onset of pain at age 14. Had multiple physiotherapy sessions over the years for managing exacerbations. Referred by GP for recent worsening of shoulder pain	*Could you please answer the following questions:* 1. What is your diagnosis for this patient? (Please provide your clinical reasoning i.e. information used to support your diagnosis, associated mechanisms of injury and alternate diagnosis excluded with justification) a. How would you classify this patient? b. Would you use an existing framework/classification system, and if so which one?
*Objective assessment* Beighton score 4/9 (bilateral elbows and knees)* Scapular dyskinesis apparent on physiological movements i.e. flexion, abduction Reluctance to elevate arm through range. Limited active range of movement end ranges of elevation with pain	
**Vignette 2** *Subjective assessment* Patient is a 14 year old male. Contact injury to left shoulder 3 days ago during a rugby match. Tackled opposing player with arm out, felt shoulder come out of place, reduced by itself. Presented to the emergency department. X-ray nothing abnormal detected. No previous shoulder injuries. Referred for rehabilitation	2. What other information/ assessment methods/ investigations would you like to have to inform your diagnosis and management plan? a.Would you consider 3D motion capture/ electromyography/ neurophysiologist referral and what information would you want?
*Objective assessment* Positive apprehension relocation test Beighton score 2/9 (bilateral knees)* Limited active range of movement in all planes with limited muscle strength compared to right	
**Vignette 3** *Subjective assessment* Patient is a 17 year old female referred for recent episode of shoulder instability and pain following collision in basketball 2 months ago. Felt shoulder pop out and in when diving for a ball on the ground. Did not attend emergency department. Unable to recall previous significant episodes of trauma. History of similar feelings previously but less severe. Unclear around the level and direction of displacement. Previous episodes associated with normal daily tasks and sports but did not affect activity or participation. Referred by GP to Physiotherapy for shoulder pain and queried shoulder dislocation. Separate referral to orthopaedic consultant pending appointment date	3. What would your management plan and prognosis for this patient be? (Please provide your clinical reasoning i.e. information used to support your management plan/prognosis) a. Is this informed by any clinical pathways or best practice guidelines?
*Objective findings* Positive apprehension relocation test Beighton score 5/9 (Bilat elbows, knees and hands flat to floor)* Full active range of movement with pain end of range elevation	

*Joints in brackets indicate where subjects received points on Beightons test i.e. where hypermobility was present
- Participants were required to individually generate ideas in response to the seed questions that accompanied each vignette (Table 1) and this was recorded in a flip chart (NB: The order of participants was randomised to ensure that the most experienced, or specialist clinician did not go first.)
- Participants were provided with opportunities to discuss any of the previously recorded responses.

Whilst it is acknowledged that there is likely to be diversity in practice, sufficient commonality is expected to be present which would allow for shared frameworks and approaches to be identified. The goal of the focus groups was therefore to (1) map current practice and identify which sources of information and frameworks are used in decision making (2) identify differences and similarities in practice alongside possible explanations, in order to see if consensus can be developed and (3) identify areas of ambiguity, which may be contributing to inaccurate diagnosis or benefit from technology based tests, as well clinicians attitudes towards these.

All focus group sessions were audio recorded, transcribed verbatim and imported into NVivo software [12]. Thematic analysis was conducted according to the stages outlined in Braune and Clarke [22]. Codes and subsequent themes were generated by a single researcher (non-clinical author) and were then verified with another researcher (clinical author). Participant (PPt) transcriptions were labelled according to anonymised participant identifiers (in the form Ppt#).

## Results

A total of 25 physiotherapists, seven males and 18 females, participated from across four different clinical sites. Clinicians were all from the UK and worked across a range of settings including primary and secondary care. Within groups, participants varied in their years of experience and levels of specialism.

Whilst some instances of similar practice were identified, the overall low levels of agreement and tacit/ semi tacit processes used in decision making could not be considered sufficient for the development of an explicit agreed minimum dataset of factors and processes used for decision making. The following themes pertaining to other components of the diagnostic process were identified:

- Variability in diagnostic processes and lack of standardised practice.
  - Differences in diagnoses and diagnostic processes.
  - Diagnostic process occurs over a long period of time.
  - Diagnostic test choices influenced by factors beyond objective markers associated with the patient injury.
  - Planning for prognosis influenced by factors beyond assessment findings.
  - Trust in staff relationships.
    - General distrust of individuals or modes of medicine used outside of the department.
    - Unity within the department.
- Knowledge and attitudes towards novel technologies for facilitating assessment and clinical decision making.
  - Lack of knowledge and rejection of 3D motion capture.

The following section provides a brief description of each theme.

### Variability in diagnostic processes and lack of standardised practice

#### Differences in diagnoses and diagnostic processes

Considerable variation was identified across focus groups regarding diagnosis and diagnostic processes, i.e. a unified structured approach could not be identified. Within and between centres, each vignette was diagnosed differently. Vignette three, for example, had 14 different diagnosis elements, some of which contradicted each other, e.g. diagnosed as traumatic in some cases and atraumatic in others. While there tended to be a general consensus for each case, these were usually over one facet of the injury, e.g. instability direction was either anterior or posterior, traumatic or atraumatic, rather than a complete diagnosis. Less than half of all participants reported being able to identify or use existing frameworks for classification. This was most clearly indicated by the following statement:

Ppt 22: ‘And then what framework do I use in classification system? Uh, [Ppt 22]’s fly by the seat of her pants framework. So I don’t, I don’t use any.’ [Vignette 1].

The few individuals who did suggest that they used a classification system typically did not record the injury using the classification system, but just kept it in mind as they moved through the diagnostic process.

#### Diagnostic process occurs over a long period of time

**Fig. 1:**
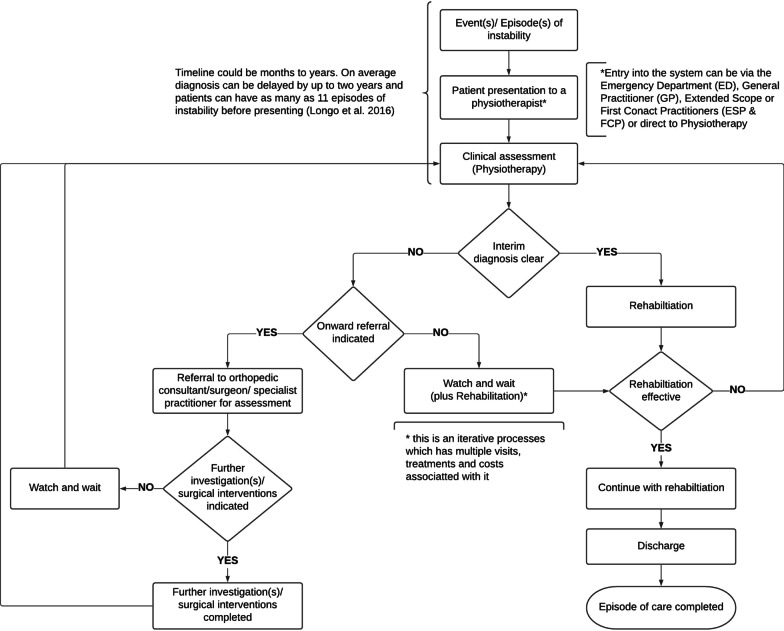
Outline of current processes for the diagnosis and management of paediatric shoulder instability

The diagnostic process was described as a period of data collection which changed and adapted as it progressed, sometimes over weeks or months, rather than within in a single appointment. This process has been outlined in Fig. 1. The participants described prioritising information collected from the physiotherapist over that recorded using technological means. Most participants only considered technology-based diagnostic tests or referrals as a potential future option if the original assessments and rehabilitation were unsuccessful. This was best displayed in the following quote:

Ppt #11: ‘we might not go for an MRI, an MRI straight away. See how they get on over the next few weeks. Um, and if they had any neurological symptoms, then look at the conduction studies’ [Vignette 2].

Diagnostic test choices influenced by factors beyond objective markers associated with the patient injury.

The regular trade-off between the idealistic and realistic when it came to carrying out tests and prescribed rehabilitation was another emergent theme of the data. Cost was the most frequently mentioned limiting factor to carrying out tests or exploring the future use of 3D motion capture.

Tied in with cost was time; both the time for carrying out the tests and general appointments but also, should referrals be needed for the patient to access the test, the time for them to move through the waiting list. This balancing act between the needs of the patient in the vignettes, and real-life factors, e.g. patient waiting times, was a common point of discussion.

Sports ability, goals of patient, and in some cases the goals of parents and family, all influenced the selection of diagnostic tests. Patients performing sport at a higher level were more likely to have referrals for technology-based objective testing in a shorter time frame than those who only played in an informal setting, summed up in the following comment:

Ppt #9:’subjective factors will, will have an influence on that, in terms of how sporty he is, and how, uh, how high-level he wants to be with that, as to whether I would pursue that further in terms of investigations in that.’[Vignette 2].

#### Planning for prognosis influenced by factors beyond assessment findings

Many of the factors identified were directly linked to the injury or how the patient recovered, such as ‘severity of pain in the initial stages’ and ‘how quickly he gets his range back’ [Ppt #18 – Vignette 2]. A wide range of additional factors were considered and prioritised in the prognosis assessments, namely age-related psychosocial influences and subjective assessment related to social situation and family relationships. Gender/Sex bias was explicit. The teenage female vignettes were linked to poorer prognosis because ‘They've got, you know, hormonal, hormonal changes going on, they've got loads going on in life.’ [Ppt #18 – Vignette 1] which was suggested to influence their likelihood to carry out the recommended rehabilitation faithfully. There were several comments about young girls’ compliance and prognosis, the following comment best sums up these discussions:

Ppt #18: ‘[teenage girls] are most likely to present with hypermobility and multi-directional instability in their shoulders. They're also the ones that most evidently, we know are most problematic to treat because they show signs of voluntary instability. They're the ones that we don't want to operate on. We don't want anybody to operate on. They're also poorly compliant, poor attenders and tricky.’ [Vignette 1].

There was some discussion surrounding psychosocial factors affecting the male vignette, but these were much less frequently mentioned and predominantly about apprehension to regain movement.

Compliance levels were deemed to influence prognosis, with participants suggesting that patients ‘will improve but [they need] to put the work in’ [PPt #17 – Vignette 1]. Sporting activity was identified as a significant part of this, with participants suggesting that patients would improve faster if they have a driving force or reason to promote positive behaviours.

Ppt #18: ‘Yeah, I'd have more concerns if she wasn’t doing any sport and have no reason to be better. But obviously, if she's still wanting to compete and do those things, then you kind of think she's got reasons to try and actually be better.’ [Vignette 1].

One individual suggested that the physiotherapists could influence compliance levels, although this was not discussed further by any of the other physiotherapists in the group.

PPt #18 ‘I think you have to be careful we don't blame them for non-compliance when actually, a lot of it comes down to how well we can make them buy in to what we're trying to get them to do.’—[Vignette 3].

The physiotherapist pre-existing knowledge and notion of whether the treatment was likely to be effective was another factor which influenced prognosis. This was particularly relevant to some groups for the injured male rugby player vignette, whereby the statistical likelihood of the shoulder injury occurring again was discussed:

Ppt 7: ‘Uh, but we know from research that given his age, and the fact that he’s male, and he’s sporty… Uh, I think he’s sporty, um, that there is likely to be a recurrence. And so, I’d have that in the back of my mind really. At that age I think it’s like over 90%, so…’ [Vignette 2].

This made up one of the few examples of literature or data supporting the answers given in the focus groups.

Trust in staff relationships.

Within the focus groups there was a general theme of trust within the department and suspicion regarding individuals who were outside of that group. This was true for both external physiotherapists and members of different departments within the hospital environment. This was expressed in a number of different ways.

General distrust of individuals or modes of medicine used outside of the department.

Distrust was common when the participants discussed medical professionals outside of their department in relation to the patient vignettes. This was expressed through active discussion of unwillingness to trust other healthcare professionals’ assessments or reports. The physiotherapists in the focus groups described additional checks which they would undertake due to them not trusting other professionals’ practices and abilities. One physiotherapist described wanting to undertake a concussion test in addition to their standard assessments.

PPt #20: ‘because I’ve had a few head injury guys that come in, like tackles or falls and things who end up being a shoulder but having been feeling quite sick and no one’s actually checked.’ [Vignette 2].

While another described wanting to repeat some of the checks provided in the vignette to confirm the data which they had been given:

PPt #20:’I’d probably redo the Beighton score as well, make sure I agree with that scoring.’

Facilitator: ‘Why would you do the Beighton score?’.

PPt #20: ‘Just so that then it’s uh, I guess it’s who’s, who’s referred. If they’ve been referred from the GP, um, how often are they doing that?’.

It is important to note, however, that where patients’ situations were judged to need or benefit from external assessment or referrals, it did not stop the participants from stating that they would refer as soon as it was needed. For example:

Ppt #7 ‘If he’s a, you know, really keen sportsman, this is his career, uh, potentially, then I’m going to refer him straight away for an assessment on the understanding that most likely we’ll be rehabbing you for three months.’

#### Unity within the department

This distrust, however, was generally absent within the groups themselves and very few members disagreed with anything which was said by their colleagues. This was verbally and structurally apparent in the construction of the group discussion. Participants tended to structure their response as a group rather than a series of individuals. When the participants responded to questions, while the first person to speak tended to answer in detail, the following responses were generally much shorter and tended to be structured as: an agreement of the former participants’ comments—often without clarifying which aspects they were agreeing with—, then an additional small detail or element which they had noticed. In some cases, though these were less common, some participants only agreed and contributed nothing else.

Groups often had one person who was a reference point for other members when they were unsure. These individuals, who tended to give much longer, detailed answers in the focus-groups, were typically senior figures within the department and with whom they consulted on a regular basis for advice regarding patients.

Facilitator: And is any of this informed by any clinical pathways or best practice guidelines?

Ppt #5: Nothing specific. I’ve always gone to Ppt #7 when I have had…

Ppt #7: [Laughs].

Ppt #5: These difficult patients and got her opinion on it.

Ppt #8: [Laughs]. I’ve asked her as well. - [Vignette 1].

Typically, these figures of reference were the only individuals who, during the focus group, alluded to the use of research or evidence to support their points.

### Knowledge and attitudes towards novel technologies for facilitating assessment and clinical decision making

#### Lack of knowledge and rejection of 3D motion capture

Participants expressed a general lack of knowledge regarding 3D motion capture which resulted in 3D motion capture being nearly completely rejected as a potential diagnostic test, best summarised by the following statement:

Ppt #10 ‘I don't know enough about it so I wouldn't want, feel confident to recommend it.’ [Vignette 1].

This was consistent with the trend or participants not looking to pursue the use of technology unless it was necessary. Only one individual suggested that they would use it in one of the scenarios. Only two individuals described personal experience of using 3D motion capture, and many others specified that their training had not covered the method at all. In some cases of discussion, participants identified potential benefits of 3D motion capture for their practice.

Rejection of 3D motion capture was justified with key concerns held against the technology. Concerns were linked to a lack of knowledge which extended to nearly every aspect of discussion including the technology itself, output it produces, the process of accessing and how it was established within the wider health service and clinical setting.

Technologically-based objective tests were described as being ‘lovely and because it would take away any question, but it doesn’t form part of our practice that we can have’  [Ppt #25 – Vignette 2], and the participants emphasised that they currently only use them when there are significant concerns or if initial attempts have failed.

Associated with the uncertainty were concerns regarding the accuracy and usability of the 3D motion capture technology, as discussed in this comment here:

PPt #20 ‘[scans] aren’t fully reliable and sensitive so I think it sort of, if we were to have something, use something like [3D motion capture], it’s how sensitive is it, how reliable is it? Um, how specific is it?’ [Vignette 1].

Other concerns raised included suitably of staff training for interpreting the results and reservations about the benefits of the additional data for the diagnostic process. Several participants showed an interest and willingness to investigate and try 3D motion capture, although in these cases participants often had inaccurate information or expectations regarding the system performance and capabilities. Participants conceptualised integrating it into their practice and derived potential benefits, best presented in this comment:

PPt #20: ‘If you had a machine or a computer system that they walked into a room and they said that my symptoms come on when I do this, they did that and then the computer says this is the problem and this is what you do, that would be amazing. [Vignette 1].

Participants also expressed a willingness to learn more about the method to make a better-informed judgement.

PPt #23: ‘It would be nice to get more experience of using it I guess.’ [Vignette 2].

Participants suggested that while there were mixed responses and concerns regarding 3D motion capture, further training and education regarding the techniques and outputs, could positively influence their decision to use this mode of analysis in the future.

## Discussion

The aim of this study was to elicit the types of information used to make clinical decisions, with the long-term goal of designing and developing appropriate decision support technologies for the assessment and management of children with shoulder instability. A fundamental requirement for development and implementation of a DDSS is to have explicit clinical decision-making processes. It is also important that DDSS are developed in partnership with the clinical end user from an early stage to facilitate use in a clinical environment [19]. Within our study it was identified that there is no common structured approach towards assessment and diagnosis, therefore limiting the ability to develop a DDSS around current practice. The data confirmed that participants were not aware of existing classification systems [23]. An agreed framework comprised of well-defined terms and precise language is important for appropriately diagnosing patients and allocating treatment. Since a notable number suggested they did not know any classification methods, discussion about reasons for not using them was not common within the focus groups. The use of research to justify the decision to not use classification systems was hardly discussed, with the majority of participants suggesting that they did not know enough about them to consider using them in practice. Whilst further education and training may raise awareness regarding existing classification systems and frameworks, it is unlikely to increase their use in clinical practice. It is possible that existing classification systems are not suitable for clinical practice, as they are often complex and not based on accurate physiological processes [24]. It is therefore important to establish an agreed language and systematic framework regarding diagnosis, before a DDSS can be implemented. Future work may look to draw upon terminologies and classification process associated with frameworks and such as the International Classification of Functioning, Disability and Health ICF [25] when mapping factors used for diagnosis in shoulder instability.

The goal of a DDSS should be to optimise processes related to reaching a formal diagnosis. This can include (1) reducing the time taken to reach formal diagnosis and (2) achieving diagnosis relatively inexpensively in a reproducible, accurate and efficient manner which may result in management that is more effective or less costly. The time taken to reach a diagnosis in a developing child with shoulder instability is excessive and existing clinical assessment methods may not be suitable for accurately identifying etiological causes during the diagnostic process. The delayed time in diagnosis in current practice may stem from, and be compounded by, the lack of an agreed framework, limitations of current clinical assessment methods and, duplication of effort e.g. repeating clinical tests between practitioners. Development of a DDSS based on current practice would likely have limited accuracy and effectiveness. Current assessment methods for clinicians such as physiotherapists or orthopaedic surgeons are based predominantly on subjective reports by the patient and measurements or specialist clinical tests performed by the clinician. Subjective reporting can be subject to recall bias [26] and clinical scales or orthopaedic tests lack sensitivity and specificity [27, 28]. Clinical decision-making processes based on these are therefore likely to be prone to error. A DDSS which incorporates technology based assessments such as 3D movement analysis may improve diagnostic accuracy of current practice that seems to operate on a trial and error-based system, informed by untested assumptions regarding physiological processes. Furthermore, the DDSS may act as a vehicle for establishing wider consensus in practice. There were no systematic processes or objective criteria for onward referral or investigations. Decisions regarding onward referral or investigations were usually driven by a failure of the patient to progress with physiotherapy, indicating this may not have been the correct treatment pathway. Given the limitations of existing assessment methods, a DDSS may be better suited signposting clinicians to additional investigations or measurement methods which could improve diagnostic accuracy. It is important however that the recommendations offered by the DDSS are reflective of the real-world clinical environments, fit within the workflow of the clinician and are perceived as useful [29]. The trade-off between perceived usefulness and effort are known to affect adoption of novel technology [30], alongside associated costs. When combined with further training and education, use of a DDSS can result in clinicians changing practice, resulting in the use of more appropriate technology for the assessment and management of the upper limb [18]. Three-dimensional motion analysis and additional imaging has been shown to improve diagnostic accuracy in shoulder instability [6], yet, several barriers to using this technology were identified, namely lack of knowledge, perceived usefulness, access and cost. These were used to justify favouring the use of physiotherapist-based tests and assessments as standard practice instead of technology-based tests:

Ppt #23: ‘But if you can identify that with a naked eye um, and then um, look to treat and change that, then actually you’re spending a lot of money videoing something that hopefully we dictate and write down.’ [Vignette 1].

Whilst cost was used to justify existing methods of practice, it is important to recognise that current diagnostic processes result in an iterative process in which patients are required to attend multiple consultations with limited chance of a positive outcome. There are significant cost implications associated with this. Development of a DDSS based on more robust measures would reduce time to diagnosis, reduce the number of visits and ultimately reduce costs. This model has been used successfully in other domains of medicine improving efficiency and patient outcomes [31, 32].

The approach of the participant in one group changed when they were able to consider an ‘ideal’ situation rather than one which reflected their work environment. Although therapists were willing to use a variety of technology and assessments, even with tests that they were not familiar with, suggesting that there is a risk that referral to specialist test can be inappropriate.

Ppt #25: ‘And again also in ideal world, you’d test everything, won’t you?’ [Vignette 1].

Clinicians were sceptical of new unfamiliar technology and individuals or modes of medicine outside of the department suggesting the importance of early stakeholder involvement in developing the DDSS [29]. Further, a DDSS may also need to support/educate the therapist with selecting appropriate tests essential to underpin accurate diagnosis [30].

Use of more objective measures, derived from technology, and used alongside an appropriate DDSS may reduce bias and the negative effects on patient outcomes. Due to the limitations of existing methods, there are inherent risks and decision making can be biased. This was evident across several of the themes identified and have been summarised in (Table 2).

**Table 2:** Themes and associated list of biases identified within the data

Theme	Possible sources of bias
*Variability in diagnostic processes and lack of standardised practice*	
Differences in diagnoses and diagnostic processes	Insufficient knowledge regarding classification systems which limited discussion regarding use of research to justify decisions in clinical practice
Diagnostic process occurs over a long period of time	Diagnostic processes involved a wide range of tests and rehabilitation methods. Physiotherapy was often perceived to be the correct starting place for patients to try ‘*a few treatment sessions before [they] started considering those other investigations’* There was a desire to see if they could enact change within the patient during a physiotherapy appointment, indicting there was a perceived role for physiotherapy
Diagnostic test choices influenced by factors beyond objective markers associated with the patient injury	Time, access and cost were perceived as barriers to additional diagnostic tests which may be beneficial to patients Barriers used to justify prioritising physiotherapist-based tests and assessments as standard practice instead of technology-based tests Prioritised the information collected from the physiotherapist over that using technological means Participants with higher levels of activity, more likely to have referrals for technology-based objective testing in a shorter time frame Tied in with this was an example where despite having similar levels of activity between male and female vignettes, the male vignette was only offered onward referral (gender bias)
Planning for prognosis is influenced by a number of factors	Diagnostic processes and decisions regarding management influenced by previous clinical experience and knowledge whether the treatment was likely to be effective Psychosocial influences were generally perceived to be only relevant for the female vignettes in a negative way
*Knowledge and attitudes towards novel technologies for facilitating assessment and clinical decision making*	
Trust in staff relationships General distrust of individuals or modes of medicine used outside of the department	Evident in discussions regarding medical professionals outside of their department in relation to the patient vignettes The physiotherapists in the focus groups described additional checks which they would undertake due to them not trusting other professionals’ practices and abilities
Trust in staff relationships Unity within the department	Very few disagreements within the departments. Verbally confirmed and structurally apparent in the construction of the group discussions Evidence of a medical hierarchy within the group and practice
Lack of knowledge and rejection of 3D motion capture	Lack of knowledge limiting participants using technology which could facilitate decision making General trend for the participants to not pursue the use of any technology unless it was perceived as necessary Perception that having additional information or data will not benefit the diagnostic process if cannot be understood or usefully integrated into the current practice

A range of factors beyond the patient injury influenced both patient assessment and prognosis. Psychosocial factors were perceived to negatively affect prognosis, mainly for the teenage female vignettes. It is important to note there were no explicit psychosocial factors stated in the vignettes. Whilst poor prognosis may be associated with psychological problems [33, 34], this is not unique to the female gender and the current assumption is founded on insufficient epidemiological data. It was also noted that the decision to refer onwards was only offered to the male vignette.

Diagnostic processes and decisions regarding management were therefore influenced by previous clinical experience and knowledge whether the treatment was likely to be effective. Overall, this may reflect clinical reasoning processes based around hypo-deductive reasoning or pattern recognition which is prone to error and may compound biases [35–37]. It is likely that some of the biases observed are common between physiotherapists and are embedded within the training at degree level, within the place of work and wider training opportunities such as continuing professional development. This was evident in the predominance of group responses and the general distrust of people outside of their group. Disagreements were rare and usually only covered one element of the diagnosis. This behaviour is reflective of the groupthink phenomenon [38] and can result in omission or exclusion of potentially important information or practices from outside the group. It is also possible that clinical practice is inherited from or influenced by more senior/experienced staff members or those to perceived to be higher in the medical hierarchy such as consultants [39]. This was also evident in the structure of the focus groups, whereby one or two key members were used as a reference point in times of uncertainty, usually a more senior figure, and practice was referenced around continuing professional development courses they attended rather than evidence-based guidelines. A DDSS may therefore be used to present clinicians with suggestions of objective criteria for assessment and management alongside differential diagnosis to be considered.

DDSS are prone to bias if the training or reference datasets used are inappropriate or if the developers of DDSS include their bias into the system [40]. Several systematic biases were identified in the assessment of paediatric shoulder instability, most notably regarding gender. In this study, the vignettes provided did not specify variations in socioeconomic backgrounds, ethnicity or other demographic information which may also be characteristics that are subject to bias. We are therefore unable to account for the impact of these features in decision making and their susceptibility to bias. An inability to appropriately understand the data used for decision making and identify sources bias can result in further propagation of bias as an inherent feature of the DDSS, negatively influencing patient outcomes, rather than alerting the clinical end user of their bias in order to mitigate against it. This would limit the ability of the DDSS to provide an objective reference source for evaluation of clinical decision making. It is recognised that this requires pathways to be established and structured around predetermined criteria and algorithmic processes which currently do not exist. Further work is therefore needed to develop these processes and evaluate what effect variations in demographic characteristics have on clinical decision making for shoulder instability.

## Limitations of the study

Despite randomisation of participants to ensure the most experienced clinician did not lead and focus groups being set up to encourage individual speech, it is acknowledged that responses were structured as a group with little disagreement and usually influenced by one or two more senior members of the group. Use of the nominal focus group technique in already established groups, in which there are hierarchies, may therefore limit generation of individual ideas and discussion or disagreement. Whilst this was done to identify common practice at separate clinical sites, future work may look to use the nominal focus group in groups comprised of different clinical sites or departments. An aim of our study was to identify the information used for clinical decision making. We were unable to identify a minimum dataset or explicitly map the processes associated with assessment and management of paediatric shoulder instability. This may be due to omission of the last stage of the nominal focus group technique in which participants vote for the most important factors. Due to the variable practice between sites and levels of agreement within sites it is unlikely that this process would have generated the desired dataset. Further work is needed to identify agreed criteria used in decision making which can be matched against explicit decision-making processes. This may be achieved or informed further by Delphi technique, semi-structured interviews or action research methodologies. Our sample was wholly comprised of physiotherapists based within a public health setting. It is recognised that patients with shoulder instability may present to and be managed by alternate healthcare professionals. It is therefore important to ensure any subsequent technology or decision support systems designed for use in clinical practice takes into consideration these factors and is transferable between services and professions.

**Fig. 2:**
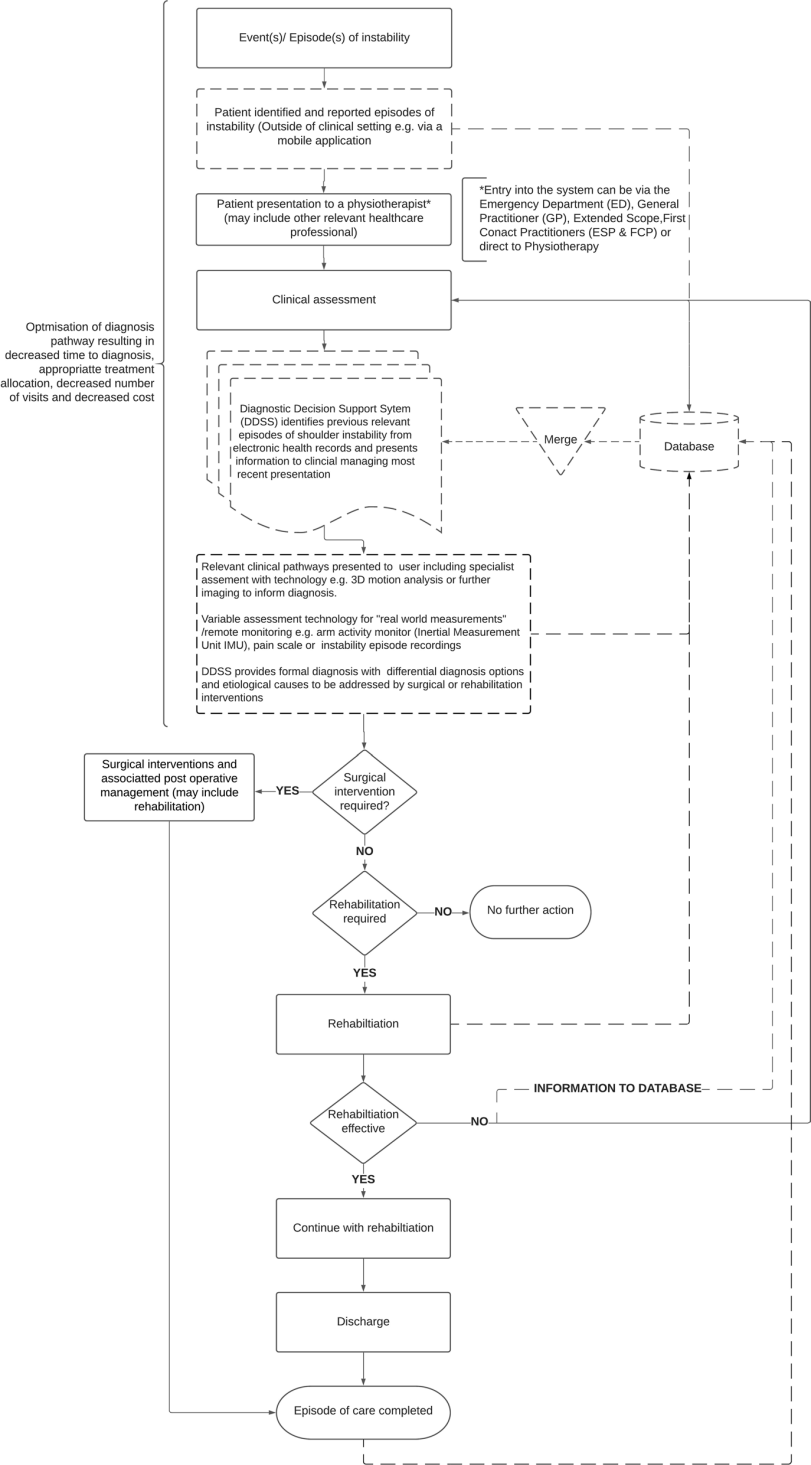
A concept map for development of a DDSS

On the basis of our findings we have produced a concept map for development of a DDSS (Fig. 2) and list of implications for development of an appropriate DDSS and associated software.

## Conclusion and implication for DDSS and software design

In order for a DDSS and associate software to be developed,

- Agreed terminology, classification and definition of terms within practice is required.
- Systematic approaches towards assessment, which can be codified and customised to match local practice are needed.
- An agreed minimum data set which is important in diagnosing paediatric shoulder instability and any area’s which would benefit from further investigations or technological assessments need to be identified to improve diagnostic accuracy.
- Agreed clinical pathways, based on established and objective criteria are required for cases in which a DDSS may be used for suggesting alternate investigations such as imaging or onward referral.
- Development of any subsequent DDSS and software will need to address the barriers around lack of knowledge, perceived usefulness, access and cost which are likely to limit the use of novel technology in practice. There is a risk that even if additional information and technology was available to clinicians, they would not use it.
- Continued early stakeholder involvement is required, particularly from senior clinicians, to ensure processes are reflective of the real-world environment, match the workflow and are perceived as useful to mitigate against rejection of the technology.
- It is important that sources of bias are identified and made explicit to minimise the propagation of bias as an inherent feature of the DSSS.

## Supplementary Information

**Additional file 1.** Clincal vignettes and seed questions (original format used for focus groups).

## Data Availability

The datasets generated and/or analysed during the current study are not publicly available due privacy/ethical restrictions but are available from the corresponding author on reasonable request.

## Footnotes

1 Hypothetical descriptions of patients developed by a specialist author (FP) and structured to reflect a range of subjective and objective features associated with shoulder instability and used to elicit decision making processes [21].
