# Supplementary material for: Implications for the design of a Diagnostic Decision Support System (DDSS) to reduce time and cost to diagnosis in paediatric shoulder instability
Source: BMC Med Inform Decis Mak. 2021 Feb 27;21:78. doi: 10.1186/s12911-021-01446-5 (PMC7912970; doi:10.1186/s12911-021-01446-5)
Supplement: Supplementary file 1 — Additional file 1. Clincal vignettes and seed questions (original format used for focus groups). [file 12911_2021_1446_MOESM1_ESM.docx]

Title page

Implications for the design of a Diagnostic Decision Support System (DDSS) to reduce time and cost to diagnosis in paediatric shoulder instability

Fraser Philp ^a*^, Alice Faux-Nightingale ^b^, Sandra Woolley ^c^, Ed de Quincey ^c^ and Anand Pandyan ^a^

^a^ School of Allied Health Professions, Keele University, Keele, United Kingdom; ^b^ School of Pharmacy and Bioengineering, Keele University, Keele, United Kingdom; ^c^ School of Computing and Mathematics, Keele University, Keele, United Kingdom;

***Corresponding author**

Dr Fraser Philp

[f.d.philp@keele.ac.uk](mailto:f.d.philp@keele.ac.uk)

Twitter: @fdphilp

ORCID: <https://orcid.org/0000-0002-8552-7869>

LinkedIn: [www.linkedin.com/in/fraserdphilp](http://www.linkedin.com/in/fraserdphilp)

**Clinical Vignettes**

**Study title**: **User centred design of decision support tools for paediatric shoulder instability**

| **Vignette 1**  **Subjective assessment**  Patient is a 16 year old female presenting with worsening right shoulder pain. Recurrent episodes of instability/ partial shoulder displacement for the last 6 years. Not sure about the direction of instability. Competitive netball and swimming since age 12 with onset of pain at age 14. Had multiple physiotherapy sessions over the years for managing exacerbations. Referred by GP for recent worsening of shoulder pain.  **Objective assessment**   - Beighton score 4/9 (bilateral elbows and knees)* - Scapular dyskinesis apparent on physiological movements i.e. flexion, abduction. - Reluctance to elevate arm through range. Limited active range of movement end ranges of elevation with pain.   ***** joints in brackets indicate where subjects received points on Beightons test i.e. where hypermobility was present |
| --- |
| ***Could you please answer the following questions:***  **1. What is your diagnosis for this patient?** (Please provide your clinical reasoning i.e. information used to support your diagnosis, associated mechanisms of injury and alternate diagnosis excluded with justification)   1. How would you classify this patient? 2. Would you use an existing framework/classification system, and if so which one? |
| **2. What other information/ assessment methods/ investigations would you like to have to inform your diagnosis and management plan?**   1. Would you consider 3D motion capture/ electromyography/ neurophysiologist referral and what information would you want? |
| **3. What would your management plan and prognosis for this patient be?** (Please provide your clinical reasoning i.e. information used to support your management plan/prognosis)   1. Is this informed by any clinical pathways or best practice guidelines? |

| **Vignette 2**  **Subjective assessment**  Patient is a 14 year old male. Contact injury to left shoulder 3 days ago during a rugby match. Tackled opposing player with arm out, felt shoulder come out of place, reduced by itself. Presented to the emergency department. X-ray nothing abnormal detected. No previous shoulder injuries. Referred for rehabilitation.  **Objective assessment**   - Positive apprehension relocation test. - Beighton score 2/9 (bilateral knees)***** - Limited active range of movement in all planes with limited muscle strength compared to right   ***** joints in brackets indicate where subjects received points on Beightons test i.e. where hypermobility was present |
| --- |
| ***Could you please answer the following questions:***  **1. What is your diagnosis for this patient?** (Please provide your clinical reasoning i.e. information used to support your diagnosis, associated mechanisms of injury and alternate diagnosis excluded with justification)   1. How would you classify this patient? 2. Would you use an existing framework/classification system, and if so which one? |
| **2. What other information/ assessment methods/ investigations would you like to have to inform your diagnosis and management plan?**   1. Would you consider 3D motion capture/ electromyography/ neurophysiologist referral and what information would you want? |
| **3. What would your management plan and prognosis for this patient be?** (Please provide your clinical reasoning i.e. information used to support your management plan/prognosis)   1. Is this informed by any clinical pathways or best practice guidelines? |

| **Vignette 3**  **Subjective assessment**  Patient is a 17 year old female referred for recent episode of shoulder instability and pain following collision in basketball 2 months ago. Felt shoulder pop out and in when diving for a ball on the ground. Did not attend emergency department. Unable to recall previous significant episodes of trauma. History of similar feelings previously but less severe. Unclear around the level and direction of displacement. Previous episodes associated with normal daily tasks and sports but did not affect activity or participation. Referred by GP to Physiotherapy for shoulder pain and queried shoulder dislocation. Separate referral to orthopaedic consultant pending appointment date.  **Objective findings**   - Positive apprehension relocation test. - Beighton score 5/9 (Bilat elbows, knees and hands flat to floor)***** - Full active range of movement with pain end of range elevation.   ***** joints in brackets indicate where subjects received points on Beightons test i.e. where hypermobility was present |
| --- |
| ***Could you please answer the following questions:***  **1. What is your diagnosis for this patient?** (Please provide your clinical reasoning i.e. information used to support your diagnosis, associated mechanisms of injury and alternate diagnosis excluded with justification)   1. How would you classify this patient? 2. Would you use an existing framework/classification system, and if so which one? |
| **2. What other information/ assessment methods/ investigations would you like to have to inform your diagnosis and management plan?**   1. Would you consider 3D motion capture/ electromyography/ neurophysiologist referral and what information would you want? |
| **3. What would your management plan and prognosis for this patient be?** (Please provide your clinical reasoning i.e. information used to support your management plan/prognosis)   1. Is this informed by any clinical pathways or best practice guidelines? |
